# Large fragment *Bst *DNA polymerase for whole genome amplification of DNA from formalin-fixed paraffin-embedded tissues

**DOI:** 10.1186/1471-2164-7-312

**Published:** 2006-12-12

**Authors:** Sarit Aviel-Ronen, Chang Qi Zhu, Bradley P Coe, Ni Liu, Spencer K Watson, Wan L Lam, Ming Sound Tsao

**Affiliations:** 1 Department of Pathology, University Health Network, 200 Elisabeth Street, Toronto, ON M5G 2C4, Canada; 2 Division of Applied Molecular Oncology, Ontario Cancer Institute/Princess Margaret Hospital, University Health Network, 610 University Avenue, Toronto, ON M5G 2M9, Canada; 3 Department of Cancer Genetics and Developmental Biology, British Columbia Cancer Research Centre, 675 West 10^th ^Avenue, Vancouver, BC V5Z 1L3, Canada; 4 Department of Laboratory Medicine and Pathobiology, Faculty of Medicine, University of Toronto, 100 College Street, Toronto ON M5G 1L5, Canada

## Abstract

**Background:**

Formalin-fixed paraffin-embedded (FFPE) tissues represent the largest source of archival biological material available for genomic studies of human cancer. Therefore, it is desirable to develop methods that enable whole genome amplification (WGA) using DNA extracted from FFPE tissues. Multiple-strand Displacement Amplification (MDA) is an isothermal method for WGA that uses the large fragment of *Bst *DNA polymerase. To date, MDA has been feasible only for genomic DNA isolated from fresh or snap-frozen tissue, and yields a representational distortion of less than threefold.

**Results:**

We amplified genomic DNA of five FFPE samples of normal human lung tissue with the large fragment of *Bst *DNA polymerase. Using quantitative PCR, the copy number of 7 genes was evaluated in both amplified and original DNA samples. Four neuroblastoma xenograft samples derived from cell lines with known N-*myc *gene copy number were also evaluated, as were 7 samples of non-small cell lung cancer (NSCLC) tumors with known *Skp2 *gene amplification. In addition, we compared the array comparative genomic hybridization (CGH)-based genome profiles of two NSCLC samples before and after *Bst *MDA. A median 990-fold amplification of DNA was achieved. The DNA amplification products had a very high molecular weight (> 23 Kb). When the gene content of the amplified samples was compared to that of the original samples, the representational distortion was limited to threefold. Array CGH genome profiles of amplified and non-amplified FFPE DNA were similar.

**Conclusion:**

Large fragment *Bst *DNA polymerase is suitable for WGA of DNA extracted from FFPE tissues, with an expected maximal representational distortion of threefold. Amplified DNA may be used for the detection of gene copy number changes by quantitative realtime PCR and genome profiling by array CGH.

## Background

With growing interest in the genomic characteristics of various human tumors and a steep increase in the availability of genomic tests for both clinical and research purposes, the amount of genomic DNA available from biological samples may limit the practicality of genomic analysis. Having been used for decades, formalin-fixed paraffin-embedded (FFPE) tissues comprise the most common form of human tissue samples archives. Therefore, it is desirable to establish a whole genome amplification (WGA) method specifically for DNA extracted from FFPE tissues. Two main approaches for WGA have been developed: thermocycling protocols and isothermal amplification methods.

Several thermocycling protocols have been used, including the degenerate oligonucleotide primed-polymerase chain reaction (DOP-PCR) [1-4], primer extension preamplification (PEP) [5-7], tagged-PCR (T-PCR) [8], and single cell comparative genomic hybridization (SCOMP), also known as linker adaptor-PCR [9,10]. What common in all these protocols are the PCR principle of temperature-dependent cyclic amplification, and the use of primers with a random sequence to allow for multiple binding sites. They differ in primer design and the sequence of temperature changes. Their amplification magnitude is a few hundredfold and the size of their DNA product ranges from 200–3000 bases. Each technique has its advantages and limitations, varying from incomplete genomic coverage to preferences for certain DNA length (*e.g*. shorter alleles in DOP-PCR [4]), and inconsistency in the magnitude of amplification and elaborated protocol (SCOMP).

Isothermal amplification methods refer to Hyperbranched Strand Displacement Amplification (HSDA), which is also known as Multiple-strand Displacement Amplification (MDA) [11-13]. MDA is based on two principles [14-16]: (1) the ability of the polymerase to cause strand-displacement, and (2) random initiation points using random primers. The 5' end of each strand is displaced by another upstream strand that is growing in the same direction. Displaced single strands are targeted by new random priming events. As more DNA is generated by strand displacement, an increasing number of random priming events occur, forming a network of hyperbranched DNA structures of high molecular weight. As the reaction proceeds, thousands or even millions of copies of the original DNA are generated. Two enzymes are capable of catalyzing MDA: *Φ29 *DNA polymerase and the large fragment of *Bacillus stearothermophilus *(*Bst *DNA polymerase, large fragment). Previous work has shown that MDA using *Bst *DNA polymerase on intact DNA (e.g. DNA isolated from fresh or snap-frozen tissue) gives rise to robust amplification with a representational distortion of less than threefold [14-16]. We have investigated the feasibility of MDA on DNA from FFPE tissue using the *Bst *DNA polymerase, and evaluated the magnitude of representational distortion using quantitative realtime PCR (QPCR) and whole genome tiling array comparative genomic hybridization (CGH) representing complete coverage of the human genome.

## Results

**Figure 1:**
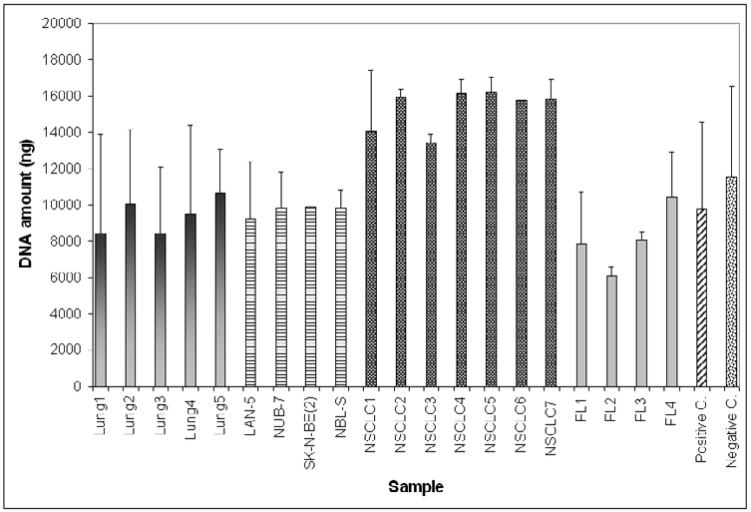
**Mean amplification of DNA by *Bst *polymerase**. All reactions started with 10 ng of target DNA. FFPE samples: Lung 1–5, neuroblastoma xenografts (LAN-5, NUB-7, SK-N-BE(2), NBL-S) and NSCLC 1–7. Intact DNA samples: FL (Frozen Lung) 1–4 and Positive C. (Control). Negative C. (Control) contained water in lieu of target DNA. For each sample the mean and SD of 2–6 independent experiments is shown.

**Figure 2:**
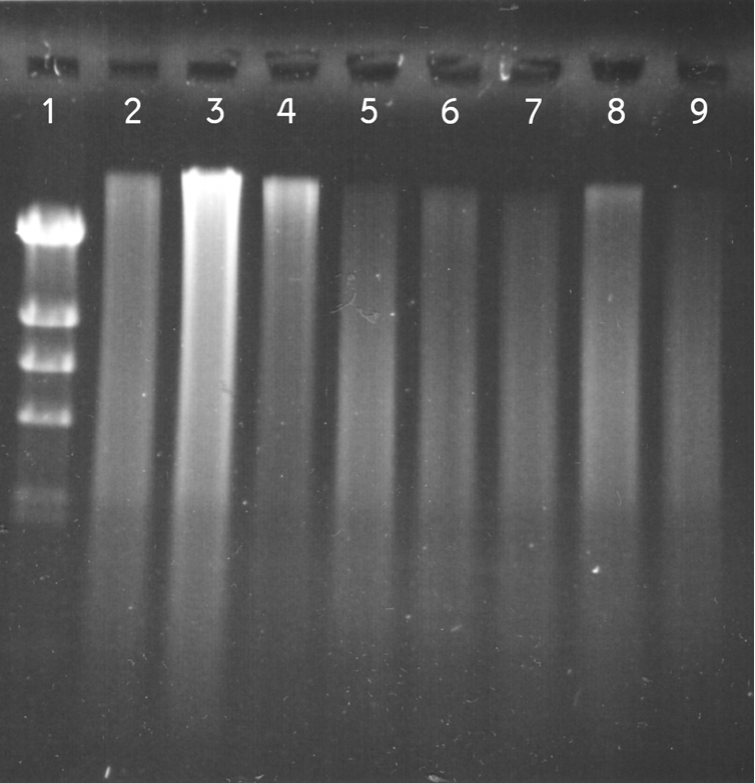
**Gel electrophoresis of *Bst *DNA polymerase amplification products**. From left to right: (1) Lambda DNA-Hind III digested ladder; FFPE samples: (2, 3) normal lung 3 & 4; (4, 5) neuroblastoma xenografts LAN-5 & SK-N-BE (2) and (6, 7) NSCLC 3 & 4; (8) Commercial DNA; (9) Negative control. Samples were analyzed in 0.5% agarose gel, stained with SYBR-green II. 10% by volume of the amplification product was used for the gel electrophoresis.

The *Bst *DNA polymerase yielded a median 990-fold (range 613–1618) of DNA amplification (Figure 1). The reaction efficiency for commercial DNA and DNA extracted from snap-frozen samples was comparable and achieved a median amplification of 803-fold (range 613 – 1043), whereas the FFPE derived DNA was amplified slightly more, with a median amplification of 1035-fold (range 839 – 1618). The amplification of the negative control also generated DNA product, which was consistent in amount to the other samples. Amplification of DNA from the human pancreatic ductal epithelium (HPDE) cell line yielded 1422 ± 310- and 1560 ± 144-fold changes without and with DNA shearing, respectively. DNA replication products were of very high molecular weight as they were larger than 23 Kb (Figure 2).

**Figure 3:**
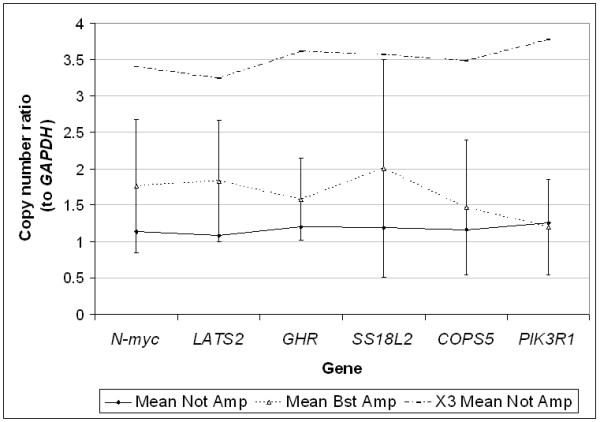
**Amplification of genes in formalin-fixed paraffin embedded normal lung tissue**. Mean values of relative amount of each gene to *GAPDH *in five samples before and after *Bst *polymerase amplification are shown. Error bars for ± 2 SD of mean values of *Bst *amplified samples are drawn. X3 Mean Not Amp is the calculated expected 3-fold representational distortion range. Gene copy numbers following *Bst *amplification resembled respective values in non-amplified samples and were within 3-fold change.

**Figure 4:**
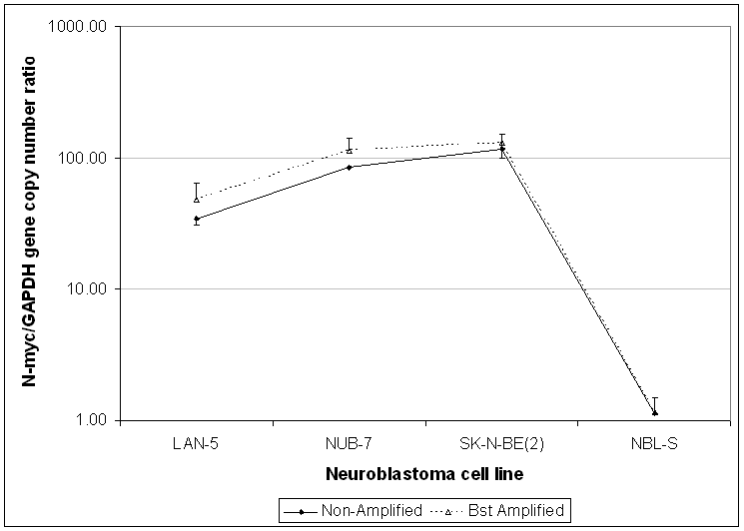
**N-*myc *gene content in *Bst *amplified vs. non-amplified neuroblastoma xenografts**. For neuroblastoma xenografts, where N-*myc *gene is highly amplified, relative gene content in *Bst *amplified samples was comparable to the respective values in non-amplified samples and the representational distortion was negligible. Note: NBL-S is a neuroblastoma cell line that lacks N-*myc *amplification and appropriately the calculated copy numbers were 1.12 ± 0.03 for non-amplified DNA and 1.14 ± 0.35 for *Bst *amplified DNA. Error bars represent SD.

**Figure 5:**
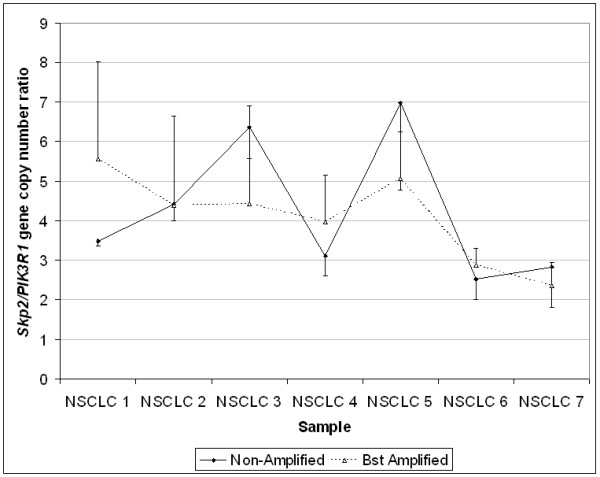
**Detection of *Skp2 *amplification in NSCLC samples following whole genome amplification by *Bst *DNA polymerase**. The ratios of *Skp2 *to *PIK3R1 *gene were maintained in *Bst *amplified vs. non-amplified NSCLC samples. Error bars represent SD.

QPCR analysis revealed similar findings in all samples, whether of normal tissue or tumoral nature, carrying known gene copy number abnormalities. All tested genes were found in all FFPE and *Bst *amplified samples, and their relative gene copy number was within 3-fold range of non-amplified samples (Figure 3 to 5). In normal lung samples, the expected copy number ratio of any given gene to *GAPDH *was 1. The values shown in Figure 3 are the average ratios of the five samples tested before and after *Bst *amplification. The average ratios of the non-amplified samples were close to 1 (range 1.08–1.26), while those following amplification were somewhat higher (range 1.20–2.00) but within 3-fold (Figure 3). For neuroblastoma xenografts where the N-*myc *gene is highly amplified, the representational distortion introduced by *Bst *amplification was negligible relative to the magnitude of gene amplification (Figure 4). For genes with low amplification levels such as the *Skp2 *gene in NSCLC (*Skp2*/*PIK3R1 *≤ 6), an increase in gene copy was detected following *Bst *amplification with a bias of up to 3-fold (Figure 5). It should be noted that in two of the non-amplified samples (NSCLC no. 6 and 7), the *Skp2*/*PIK3R1 *ratio was lower than 3, and therefore within the bias range. Nevertheless, it was detectable after *Bst *amplification.

In the negative control, the amplification reaction produced substantial amounts of DNA. However, no genes were ever detected by QPCR, indicating that the measured product was the result of a spurious amplification of the primers.

**Figure 6:**
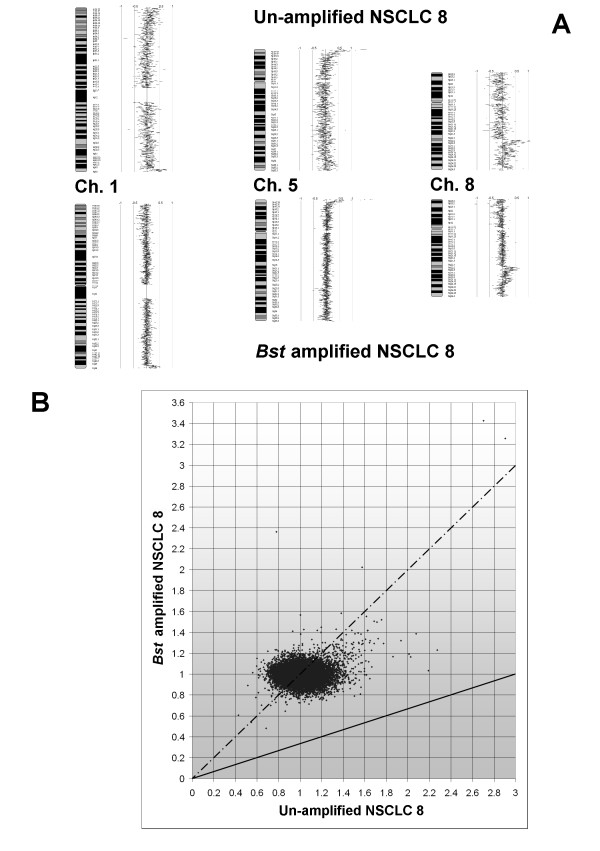
**Array CGH of NSCLC before and after *Bst *amplification**. (A) Data for chromosomes 1, 5 & 8 are displayed as a karyotype diagram with values corresponding to log_2 _ratio of Cy5/Cy3 spot signal (SeeGH v1.6). The genome profile following *Bst *amplification was similar to the profile of the original sample. Clones with log_2 _ratio <0.5 at 1p escaped detection following *Bst *amplification. (B) Scatter diagram comparing ratio of Cy5/Cy3 spot signal of NSCLC 8 before and after *Bst *amplification. Solid line: expected 3 fold representational distortion. Dashed line: desired (1:1) ratio of ideal WGA devoid of representational distortion. Comparison of the signal ratio for NSCLC 8 before and after *Bst *amplification shows it is near ideal (1:1) ratio.

Array CGH genome profiles of the *Bst*-amplified DNA from two NSCLC tumours were similar to profiles obtained using their respective non-amplified DNA (Figure 6A). Hybridization of *Bst*-amplified samples against *Bst*-amplified reference DNA allowed for the identification of genomic changes that were below 3-fold change. For each of the array CGH clones, the ratio of sample to reference signal defines the changes in gene content of a given tumour. This ratio should not change for each clone when an optimal WGA method is used. Figure 6B illustrates that the correlation of such ratios was near ideal (1:1) between the un-amplified and *Bst*-amplified DNA from NSCLC 8; similar finding was found in NSCLC 9. The four CGH arrays had variable quality; therefore a different number of human bacterial artificial chromosome (hBAC) clones was evaluated for each pair of arrays hybridized to non-amplified and amplified DNA (Table 2). Both NSCLC samples had normal gene content in more than half of the hBAC clones, and detected changes were of low gene-dosage. Analysis using the aCGH-Smooth software showed that 58.3–84.3% of the clones had a matching call (normal gene content/amplification/deletion) following *Bst *amplification. The percentage of matching clones correlated with the level of genomic changes: the higher the un-amplified sample/reference ratio was (especially > 3), the more likely it was to be detected correctly following *Bst *amplification. NSCLC 8, which had higher level of genomic gains compared to NSCLC 9 (as reflected by the highest ratio of un-amplified sample/reference of 2.9 vs. 2.1), also exhibited a higher percentage of hBAC clones with matching profiles (84.3% vs. 58.3% respectively). The level of disagreement between the paired arrays was expressed by the number of clones that changed after *Bst *amplification. Both NSCLC samples had more amplified than deleted clones. Likewise, more amplified than deleted clones were undetected after *Bst *amplification. However, based on the percentage of originally amplified/deleted clones, gene deletions were more prone to escape detection following *Bst *MDA (95.83–100%) compared to gene amplifications (67.22–92.31%).

In contrast to the successful *Bst *MDA of FFPE DNA, repeated attempts to amplify FFPE DNA using *Φ29 *DNA polymerase failed. Although the latter yielded 568 ± 342-fold DNA amplification and the reaction product was visible on a gel, QPCR of genes successfully validated on *Bst *MDA products consistently failed on *Φ29 *DNA polymerase WGA products. To rule out the inadequacy of FFPE DNA or of the *Φ29 *DNA polymerase reaction, we repeated the QPCR reactions on non-amplified FFPE DNA and DNA from frozen tissue before and after *Φ29 *DNA polymerase amplification and were able to detect all genes.

## Discussion

Lage and Dean *et al *[14,15] reported that MDA demonstrates high-amplification potential and excellent loci representation with less than 3-fold bias. Our study showed for the first time that *Bst *MDA is feasible and reliable for WGA even on FFPE DNA. We have demonstrated that in three groups of FFPE samples (normal lung tissue, neuroblastoma xenografts and NSCLC), the relative content of different genes was maintained following the amplification. If a bias existed, it was limited to a 3-fold change. We deliberately monitored the content of genes that are located on separate, unrelated regions of the genome to provide a good estimation of the overall amplification process. Array CGH data further supports the adequacy of *Bst *MDA on FFPE DNA. Hybridization against *Bst*-amplified reference DNA allows detection of genomic changes that are even below 3-fold change.

In our study, median amplification ranged from 803- to 1035-fold, and was higher for FFPE samples than for intact DNA isolated from snap-frozen tissue or commercial DNA. This is more than the 250-fold reported previously [14]. The discrepancy might be attributed to the method used for quantitation of the template and products (PicoGreen DNA quantitation vs. NanoDrop spectrophotometry). The wide range in amplification yield may be due to variability in DNA quality and tissue fixation. A possible explanation for the higher yield in FFPE compared to intact DNA could be the preferential amplification of shorter DNA fragments by the *Bst *polymerase [17,18]. However, the partial shearing of intact genomic DNA did not result in a significant change of the amplification yield. Yet, our results contradict previous mathematically-based predictions of a lower yield with sheared DNA [14].

Our QPCR analysis of gene copy content of FFPE samples following *Bst *amplification demonstrated up to a 3-fold change with respect to the non-amplified samples, fitting earlier reports of up to 3-fold representational bias [14,15]. To compare the bias resulting from various WGA methods is challenging, as the reported characteristics of each method depend on the initial amount of DNA template used, as well as on the application under investigation. The representational bias can be implied from the reported range of efficiency rates for amplification of DNA sequences of several microsatellite loci. When performed in single cells, DOP-PCR efficiency rate ranged from 0–10% and was inferior to PEP-PCR and improved PEP-PCR (I-PEP-PCR) that ranged from 0–20% and 20–50%, respectively [6]. Dean *et al *[15] specifically compared the representational bias of three WGA methods and reported a 10^3^–10^6 ^representational bias with DOP-PCR, 10^2^–10^4 ^bias with PEP-PCR, and less than a 3-fold bias with MDA, which remained almost constant between 100- to 100,000-fold amplification.

It is known that MDA by either *Φ29 *or *Bst *DNA polymerases gives products even in the absence of DNA template. This is thought to result from spurious amplification of the primers. Lage *et al *[14] reported that background DNA synthesis was completely suppressed when modified primers with two 5'-nitroindole (universal base) residues were used. We were unable to eliminate primer amplification reactions despite the use of modified primers. However, this spurious primer amplification appears to be tolerable, since tested genes were consistently detected by QPCR in all the *Bst*-amplified samples but not in the amplified negative control.

The two enzymes used for MDA, *Φ29 *DNA polymerase and the large fragment of *Bacillus stearothermophilus*, have distinct qualities. Where as *Bst *DNA polymerase is devoid of the 3'→5' exonuclease, *Φ29 *DNA polymerase holds this proofreading activity. Therefore, *Φ29 *DNA polymerase has a lower error rate, more efficient amplification reactions and appears to be more suitable for additional sequencing studies. However, *Bst *DNA polymerase seemed to demonstrate greater fidelity for copy number than *Φ29 *DNA polymerase. One reason is the significantly reduced activity of *Φ29 *DNA polymerase at the telomeres. In contrast, *Bst *DNA polymerase can switch templates and is therefore less affected by the proximity of genes to the telomere [14]. Thus, *Bst *MDA may be a better choice for WGA in array-CGH studies. It was suggested that MDA (by either *Φ29 *or by large fragment *Bst *DNA polymerase) is appropriate for array-CGH from the point of view of both sequence fidelity and representation distortion with the following guidelines. First, amplification should be < 1000-fold to keep the representation distortion less than threefold [14-16]; second, only gene copy number changes that are minimally threefold can be reliably detected [14], and finally, array-CGH study design should include amplification of the sample of interest and reference genomic DNA under identical conditions to minimize biases [14]. Based on our results, we also recommend that these guidelines should be adopted whenever *Bst *MDA is followed by QPCR for gene copy number evaluation. Copy number changes detected following *Bst *amplification are reliable only if they are higher than the 3-fold representational distortion range. Thus, it is expected that high copy number changes (*e.g*. N-*myc *in neuroblastoma xenografts) will be easily detected, since even with 3-fold amplification bias, the change in copy number is conspicuous compared to normal gene content. Although low copy number changes (*e.g. Skp2 *in NSCLC) are also detectable, the difference in copy number relative to normal gene content may be attenuated with 3-fold representation distortion.

Our array CGH results using FFPE DNA are remarkably similar to results previously obtained on intact DNA. With 1000-cell experiments, Lage *et al *reported a concordance between the amplified and non-amplified DNA array CGH of 53.6–83.3% [14]. We found concordance of 58.3–84.3% when *Bst *amplification was applied to 10 ng of DNA starting material. Lage stated that altered loci with relatively high gene-dosage alterations were detected with high reproducibility. Likewise, we observed that gene amplifications were consistently detected, even when smaller than 3-fold, yet the detection sensitivity correlated with the level of genomic changes. We noted that gene deletions were prone to be missed following *Bst *amplification and similar findings can be seen in the data presented by Lage *et al*. Array properties, the quality of the amplified DNA, the length of deleted areas and the low level of gene content change in deletions, which is strongly affected by the amplification representation bias introduced by MDA, all contribute to the reduced detection ability of deletions following *Bst *amplification. Altogether, we found that *Bst *MDA on FFPE DNA is reliable for following genome profiling by array CGH, in particular for the detection of gene amplification.

Prior to 2003, there were very few studies on the use of MDA compared to the widely reported PCR-based WGA [19]. However, there has recently been a growing interest in the method, as reflected by the increasing number of papers published during the past two years. MDA was reported to succeed in a variety of applications, including sequencing [20,21], microsatellite marker analysis [22,23], SNP analysis [24,25], genotyping [26] and array-CGH [14,27]. All these studies used the *Φ*29 DNA polymerase, and all but one [28] used DNA isolated from fresh or snap-frozen tissue samples. Like others [26], we have failed in our attempts to amplify FFPE samples with the *Φ*29 polymerase. Although the reaction yielded DNA that was 500-fold greater than the initial amount, no genes could be detected by QPCR and results were consistent with a spurious amplification of the primers. On the other hand, Wang *et al *[28] reported a successful *Φ*29 amplification of FFPE-derived DNA after the addition of a preliminary restriction enzyme fragmentation step. The modified protocol was named Restriction and Circularization Aided Rolling Circle Amplification (RCA-RCA). Our work is the first to describe the use of the large fragment of *Bst *DNA polymerase for WGA of FFPE DNA.

Aside from PCR-based techniques and MDA, a novel approach to WGA is the T7-based linear amplification of DNA (TLAD). TLAD appears free of sequence and length-dependent biases, and is thus applicable to FFPE DNA. However, this technique requires purification following each step and is therefore laborious and vulnerable to sample loss [19,29].

## Conclusion

We have shown that the large fragment of *Bst *DNA polymerase is suitable for WGA of DNA extracted from FFPE tissues, with an expected representational distortion of up to threefold. Amplified DNA may be used for the detection of gene copy number changes by QPCR and genome profiling by array CGH.

The expected application, magnitude of findings and the limits of the method are factors that need to be considered when WGA is chosen. The virtues of *Bst *DNA polymerase use for WGA should be emphasized: the method is efficient, technically easy, gives high yield and is suitable for FFPE DNA. We believe that in time, *Bst *DNA amplification will become part of routine molecular laboratory work for research and clinical purposes.

## Methods

### Tissue materials and genes

The University Health Network Research Ethics Board has approved this study protocol. Tissues were obtained from NSCLC patients who underwent tumour resection at the University Health Network. Snap-frozen tissues were banked within 30 min after resection. Archival paraffin embedded tissues were 4 to10 years old (from 1994–2000); they were fixed in 10% buffered formalin and processed according to routine pathology departmental protocols. DNA was extracted from four snap-frozen normal lung tissue samples and five FFPE normal lung tissues. Commercial human male DNA (Novagen, Madison, WI) served as a positive control, and water in lieu of target DNA served as the negative control. Gene copy number both prior to and following *Bst *amplification was assayed by QPCR for *GAPDH *(12p13), N-*myc *(2p24.1), *SS18L2 *(3p21), *GHR *(5p12-13), *PIK3R1 *(5q13.1), *COPS5 *(8q13.1) and *LATS2 *(13q11-12). Four xenografts of neuroblastoma cell lines with known N-*myc *gene copy number including LAN-5 [30,31], NUB-7 [32,33], SK-N-BE (2) [34,35] and NBL-S [36] were also studied. Seven non-small cell lung cancer (NSCLC) samples with known *Skp2 *gene amplification [37] were also similarly evaluated. Tumor cells from the xenograft and NSCLC samples were enriched by manual microdissection from sections stained by toluidine blue. To compare the amplification yield in intact and fragmented DNA, we used DNA from three clones of the normal human pancreatic ductal epithelium (HPDE) cell line. DNA was sheared by sonication to strands of less than 4 Kb.

### DNA extraction from FFPE tissue

Tissue sections 5 μm thick were de-waxed in toluene with vibration for 10 seconds, followed by 2 ethanol washes (with 100% and 75% ethanol) after brief vortexing. Digestion with 0.5 mg/ml Proteinase K (Roche, Laval, QC, Canada) in digestion buffer (50 mM KCl, 10 mM Tris-HCl (pH 8.3), 0.1 mg/mL gelatin, 0.45% Igepal CA-630 and 0.45% Tween) was carried out over-night, and was inactivated in 100°C for 10 minutes. The samples were purified by Phase Lock Gel (Eppendorf, Westbury, NY) with Phenol:Chloroform:Isoamyl Alcohol 25:24:1, and precipitated in 3 volumes of 0.1 M NaOAc in 95% ethanol overnight at -20°C. DNA was re-suspended in water at 37°C for 1 hour.

### *Bst *DNA polymerase and *Φ29 *DNA polymerase MDA

DNA was amplified by the large fragment of *Bst *DNA polymerase according to Lage *et al *[14]. Briefly, 10 ng of DNA were mixed with 1.5 μL of 10× ThermoPol buffer (New England Biolabs, Ipswich, MA) and primers (random 7-mers with an additional two nitroindole residues at the 5' end and a phosphorothiate linkage at the 3' end) at a concentration of 100 μM in 15 μL. DNA was denaturated at 96°C for 2 minutes, cooled at room temperature for 10 minutes and then placed on ice. The reaction mixture was then brought up to 50 μL with 400 μM dNTPs in 1× ThermoPol buffer, 0.35 units/μL *Bst *DNA polymerase, large fragment (New England Biolabs, Ipswich, MA) and 4% final concentration of DMSO. T4 gene 32 protein (Amersham Biosciences, Piscataway, NJ) was added to the reaction in final concentration of 30 ng/μL. The reaction was carried out at 50°C for 6 hours and inactivated at 80°C for 15 minutes. Amplified samples were purified using Phase Lock Gel (Eppendorf, Westbury, NY) with Phenol:Chloroform:Isoamyl Alcohol 25:24:1 and precipitated in 3 volumes of 0.1 M NaOAc in 95% ethanol overnight at -20°C. Amplified DNA was re-suspended in water at 37°C for 1 hour and the concentration was measured by NanoDrop (NanoDrop Technologies, Wilmington, DE). 10% by volume of the amplification products were analyzed on 0.5% alkaline agarose gels stained with SYBR-green II (Molecular Probes, Eugene, OR). For *Φ29 *DNA polymerase, we used the GenomiPhi DNA amplification kit (GE Healthcare, Piscataway, NJ) according to the manufacturer's protocol.

### Gene copy number evaluation based on quantitative realtime PCR

QPCR was performed using the SYBR Green technique in a Mx3000P^® ^QPCR System (Stratagene, Cedar Creek, TX). Primers were designed using Primer Express software v1.5 [38] (Applied Biosystems, Foster City, CA) and tested for their specificity by alignment using the BLASTN program, followed by dissociation curve and primer efficiency tests. The target amplicons were 61-144 bp in intron 1 of each gene. The sequences of the genomic primers used for subsequent QPCR assays are listed in Table 1. Five ng of non-amplified target DNA was used in each QPCR reaction, compared to 50 ng of *Bst*-amplified DNA. Gene copy number was normalized to that of *GAPDH*, and pooled DNA from five FFPE normal lung samples was used as a calibrator. Relative gene copy number was calculated using the formula: 2^-ΔΔCt ^[38,39]. In NSCLC samples, *Skp2 *was normalized against *PIK3R1 *(instead of *GAPDH*) since *PIK3R1 *was previously reported to show no amplification in NSCLC, unlike *GAPDH *[37,40].

### Array CGH of non-amplified and *Bst *amplified samples

Paired un-amplified and *Bst-*amplified DNA from two NSCLC tumours were studied and compared for genomic profile changes using whole genome array CGH. We used the "27 K" high-density hBAC Sub Megabase Resolution Tiling (SMRT) set array CGH (BCCRC, Vancouver, BC), which contains two replicates for each clone and has a resolution of 70–80 Kb. The experiments were performed as previously described [41,42]. Briefly, 400 ng of both sample and reference male genomic DNA (Novagen, Mississauga, ON, Canada) were labeled with Cyanine-5 and Cyanine-3 dCTPs (PerkinElmer, Woodbridge, ON, Canada), respectively. *Bst-*amplified samples were hybridized against *Bst*-amplified reference DNA. All MDA DNA was sheared by sonication into 3–4 Kb prior to labeling. Following hybridization, arrays images were captured by the charge-coupled device (CCD) scanner system (PerkinElmer, Wellesley, MA, USA) and analyzed with SoftWoRx Tracker (Applied Precision, Issaquah, WA, USA). Data were normalized using a three-step normalization framework [43]; replicate data points with a log_2 _ratio that exceeded a standard deviation of 0.075 were excluded. Data were analyzed with SeeGH v1.6 [44] and aCGH-Smooth [45] software with the Lambda and breakpoint per chromosome settings set to 6.75 and 100, respectively. This analysis defines chromosomal breakpoints and identifies chromosomal sections with abnormal gene content, namely areas of gene amplification or deletion. As the chromosomal location of each hBAC clone is known, the breakpoint information can be presented at the clone level where each clone can be defined as having either amplification, normal content or deletion. Each of the arrays was independently analyzed and evaluable clones before and after *Bst *amplification were compared. The concordance between paired arrays was indicated by the number and percentage of clones with matching profiles, while discordance was shown as the number and percentage of clones that changed after *Bst *amplification (Table 2). The concordance between paired arrays was also demonstrated by the correlation of the sample to reference signal ratio between un-amplified and *Bst*-amplified DNA (Figure 6B).

## Abbreviations

CGH – comparative genomic hybridization, FFPE – formalin-fixed paraffin-embedded, hBAC – human bacterial artificial chromosome, MDA – multiple-strand displacement amplification, NSCLC – non-small cell lung cancer, PCR – polymerase chain reaction, QPCR – quantitative realtime PCR, WGA – whole genome amplification.

## Authors' contributions

SAR carried out the molecular genetic studies, performed the statistical analysis, contributed to the conception of the study and its design and drafted the manuscript. CQZ participated in the molecular genetic studies and contributed to the statistical analysis. BPC participated in statistical analysis. NL and SKW participated in molecular genetic studies. WLL contributed to the conception and design of the study. MST conceived of the study, participated in its design and helped to draft the manuscript. All authors read and approved the final manuscript.

## Figures and Tables

**Table 1:** Primers sequences

**Gene**	**Amplicon**	**Forward primer**	**Reverse primer**
GAPD	125 bp	5'-GGTAAGGAGATGCTGCATTCG-3'	5'-CGCCCAATACGACCAAATCTAA-3'
NMYC	111 bp	5'-CGCAAAAGCCACCTCTCATTA-3'	5'-TCCAGCAGATGCCACATAAGG-3'
SS18L2	127 bp	5'GTAGGGATGAGGTCTCCCTTTGT-3'	5'-GAAATGCGGAGCTGGTGTG-3'
GHR	120 bp	5'-GACTGGCCACTTAGCTGTCTTTG-3'	5'-GGAGTCCTTTGAGTAGCAGCAACT-3'
PIK3R1	144 bp	5'-TCATTTGTGGGATGACTTAGATTTG-3'	5'-AAAGTTGACAGTCCTGAATATTTTTAATATATAAA-3'
COPS5	61 bp	5'-TCGACATGCACCTTGTTTGG-3'	5'-TGAAAACAGCTGCAATCCCC-3'
LATS2	101 bp	5'-GAGTCAGGGAACCTGGCTTTAA-3'	5'-ATATGACTCTTCGGCAGCTGC-3'
SKP2	102 bp	5'-GGGTACCATCTGGCACGATT-3'	5'-GATACTGCTATTCTGAAAGTCTTTTTCTTC-3'

**Table 2:** Comparison of array CGH genome profiles before and after *Bst *amplification

		Number of clones	Normal gene content	Gene amplification	Gene deletion
NSCLC 8 (2.9*)					

Before *Bst *MDA		16277	12779	3450	48

Comparing after to before MDA	Matching clones	13725 **(84.3%)**	12592 (91.75%)	1131 (8.24%)	2 (<0.01%)
	Clones that changed	2552 (15.7%)	187 (7.3%)	2319 (90.9%)	46 (1.8%)
	% of abnormal clones before MDA			67.22%	95.83%

NSCLC 9 (2.1*)					

Before *Bst *MDA		23942	13538	9483	922

Comparing after to before MDA	Matching clones	13960 **(58.3%)**	13231 (94.8%)	729 (5.2%)	0 (0%)
	Clones that changed	9982 (41.7%)	307 (3.1%)	8754 (87.7%)	922 (9.2%)
	% of abnormal clones before MDA			92.31%	100%

Data from each of the four array CGH experiments (NSCLC 8 & 9 before and after *Bst *amplification) was normalized and replicate data points with standard deviation of log_2 _ratio > 0.075 were excluded. Following, data was analyzed with aCGH-Smooth software, which identifies breakpoints and areas of gene amplification and deletion. The numbers presented refer to clones that were evaluable both before and after *Bst *amplification for each of the tumors. * Highest ratio of un-amplified sample/reference.
